# Integrative Analysis of a Pyroptosis-Related Signature of Clinical and Biological Value in Multiple Myeloma

**DOI:** 10.3389/fonc.2022.845074

**Published:** 2022-02-28

**Authors:** Huizhong Wang, Ruonan Shao, Shujing Lu, Shenrui Bai, Bibo Fu, Renchun Lai, Yue Lu

**Affiliations:** ^1^ Sun Yat-sen University Cancer Center, State Key Laboratory of Oncology in South China, Collaborative Innovation Center for Cancer Medicine, Guangzhou, China; ^2^ Department of Hematologic Oncology, Sun Yat-sen University Cancer Center, Guangzhou, China; ^3^ Department of Anesthesiology, Sun Yat-sen University Cancer Center, Guangzhou, China

**Keywords:** multiple myeloma, pyroptosis, nomogram, prognostic model, tumor microenvironment

## Abstract

**Purpose:**

Pyroptosis is an inflammation-based programmed cell death that holds great potential as a novel cancer therapeutic target in patients with multiple myeloma (MM). However, thus far, the function of pyroptosis-related genes (PRGs) in MM and their prognostic relevance remains undetermined.

**Methods:**

The model was established by the LASSO analysis, based on the Gene Expression Omnibus (GEO) dabatase, and its efficacy was verified using two external datasets. The model’s predictive ability was assessed by the Kaplan-Meier survival and time-dependent receiver operating characteristic (ROC) curves. Finally, a nomogram was established for clinical application. We also confirmed the validity of our model using specimens and *in vitro* experiments.

**Results:**

We established an 11-PRG signature profile, and verified its efficacy using two validation cohorts (VCs). In both cohorts, patients were separated into two subpopulations, according to their median risk scores (RS). Our analysis revealed that high-risk (HR) patients experienced considerably lower overall survival (OS), compared to the low-risk (LR) patients. Using functional enrichment and immune infiltration analyses, we demonstrated that the immunologic status was strongly related to RS. Furthermore, using a pyroptosis inhibitor Q-VD-OPh, we revealed that MM cell proliferation and progression was drastically suppressed and the doxorubicin (DOX)-induced apoptosis was reversed.

**Conclusion:**

Based on our analysis, pyroptosis not only serves as a measure of MM treatment efficiency and patient prognosis, but is also a possible target for anti-MM therapy.

## Introduction

Multiple myeloma (MM) is a specific tumor caused by the heterogeneous clonal proliferation of plasma cells. Its incidence is relatively high, and accounts for approximately more than 17% of hematologic malignancies, and it is increasing year by year (1). With the continuous emergence of novel treatment methods, the survival time of MM patients continues to extend, but it still remains an incurable disease (2). Advancements in molecular technology promoted the widespread use of individualized, biological markers in the diagnosis and therapy of MM (3). In response to the needs of individualized treatment, the development of new biomarkers and effective models that predict MM prognosis, along with the detection of novel targets of MM therapy hold much clinical significance.

Pyroptosis, or caspase 1-dependent cell death is innately inflammatory, and is related to the stimulation of multiple pathological factors involved in cell death (4, 5). Pyroptosis is triggered by specific inflammasomes that depend on gasdermin D (GSDMD) cleavage and stimulation of inactive cytokines (6, 7). It is generally considered that the relationship between pyroptosis and tumor development is extremely complicated. On one hand, being a programmed death process, pyroptosis inhibits tumor development. Alternately, since pyroptosis activates pro-inflammatory cytokines, it provides a suitable tumor microenvironment (TME) for accelerated growth, complete with nutrition and growth inducers (8). Emerging studies reported on the significance of pyroptosis in tumor cell proliferation, invasion and metastasis, which ultimately affects patient prognosis (9, 10). In addition, its release of massive amounts of inflammatory cytokines induced a potent immune response, which ultimately reshapes TME (11). Till date, few studies demonstrated a link between pyroptosis and MM (12, 13). Moreover, the significance of pyroptosis-related genes (PRGs) in MM prognosis is undetermined.

Herein, we explored the significance of a PRG-based signature in MM patients by examining PRG expression and related clinical information within the Gene Expression Omnibus (GEO) database, using bioinformatics analysis. The predictive value of our model was further validated using external GEO cohorts, and the expression of PRGs within our model was validated by qt-PCR analysis. In addition, we evaluated the associated regulatory axis in MM using *in vitro* experimentation. Based on our results, PRGs hold great potential in serving as prognostic indicators or therapeutic targets for anti-MM therapy.

## Materials and Methods

### Sources of Publicly Datasets

**Figure 1:**
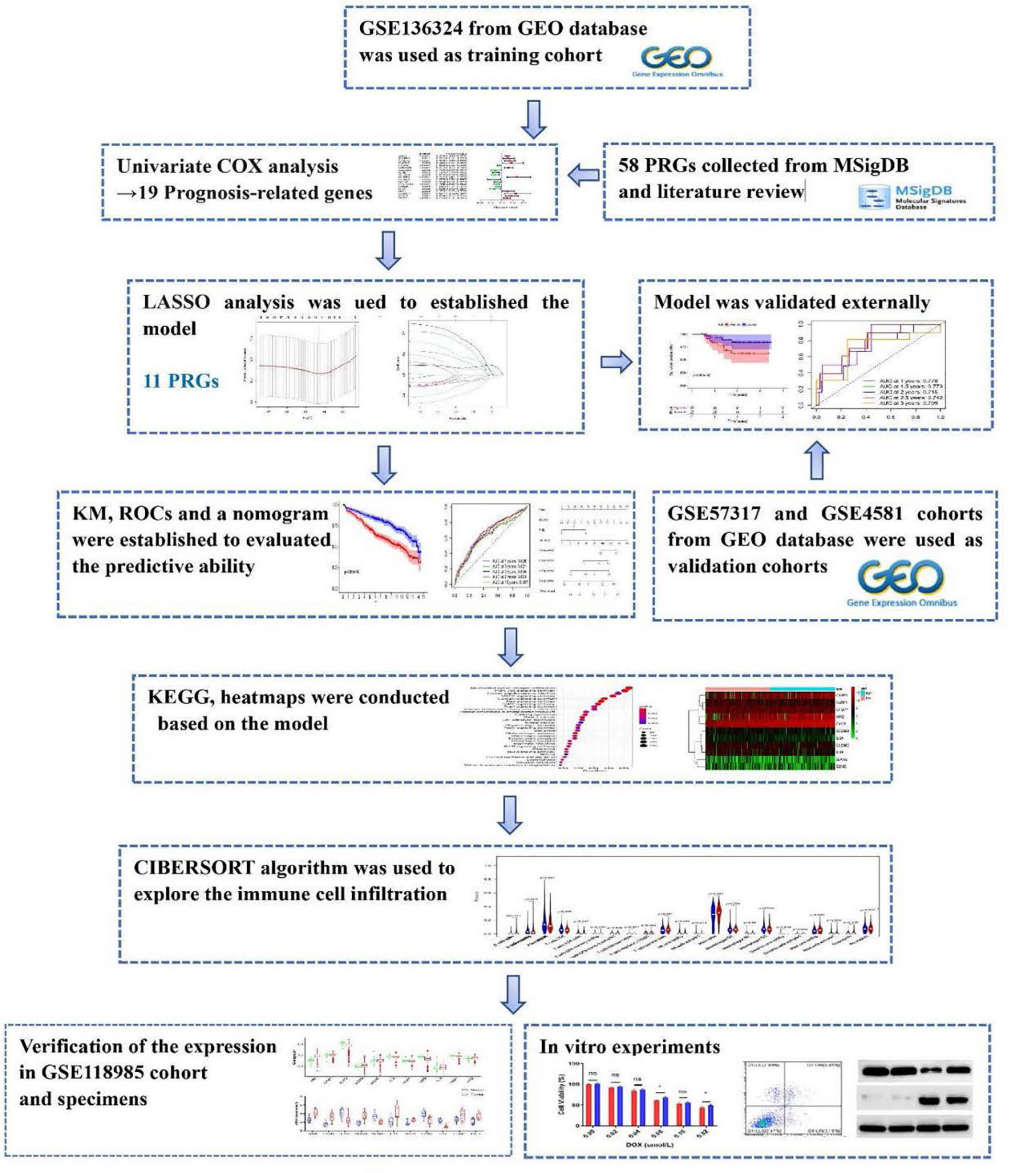
Flow chart of the evaluation and selection of IRGPI.

The transcript levels and matched clinical information of three MM populations were collected from the GEO (http://www.ncbi.nlm.nih.gov/geo/) database, namely, GSE136324, GSE57317, and GSE4581 cohorts. Samples were excluded if corresponding survival data were missing. The comprehensive summary of patient characteristics was presented in **Table S1**. The relative gene expression was normalized using the “limma” R package. The PRGs were acquired from the REACTOME_PYROPTOSIS and GOBP_PYROPTOSIS gene sets from the MSigDB database (https://www.gseamsigdb.org/gsea/msigdb/), as well as prior reviews (14). A workflow chart describing the samples utilized at each stage of analysis is presented in **Figure 1**. The datasets of this study are publicly available from the GEO database.

### Generation and Confirmation of a Prognostic Model

**Figure 2:**
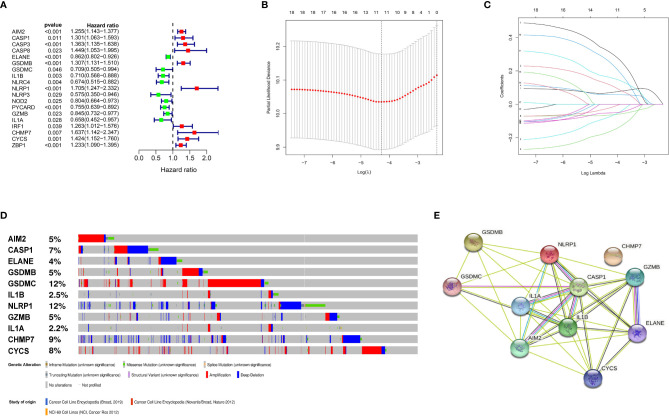
Construction and validation of the prognostic model. **(A)** Forest plots showing the results of the univariate Cox analysis between gene expression and OS. **(B)** 100000 bootstrap replicates by lasso regression analysis for variable selection. **(C)** LASSO coefficients of IRGs. **(D)** Genetic alterations of the 11 IRGs in CCLE. **(E)** The protein-protein interactions between the model related proteins and the other proteins.

**Figure 3:**
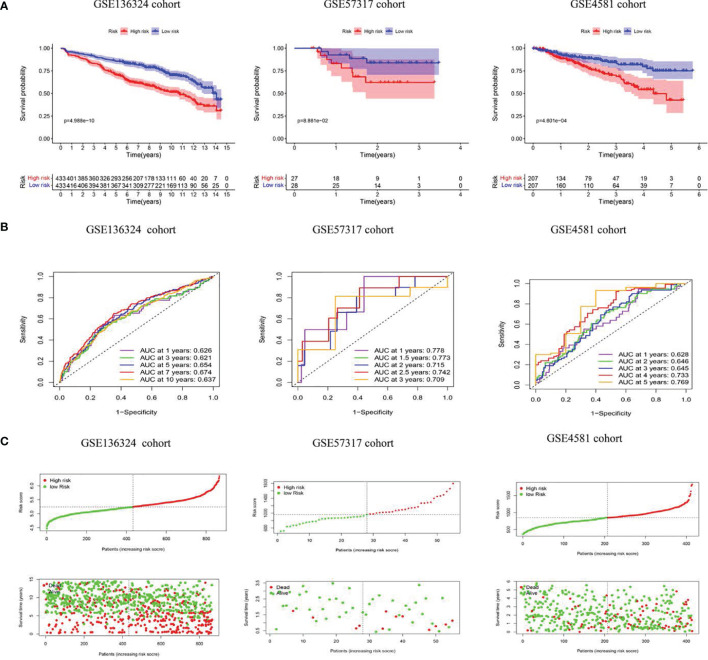
The model predicts survival of MM patients in LR and HR. **(A)** KM survival analyses, **(B)** time-dependent ROC curves analyses in all cohorts, **(C)** Risk score distribution and survival status.

We selected the GSE136324 cohort as the training cohort (TC). Using univariate analysis, we identified PRGs that were strongly correlated with patient prognosis (*p* < .05). The least absolute shrinkage and selection operator (LASSO) analysis was employed to obtain an optimal PRG weighting coefficient (15). Subsequently, we performed a 10-fold cross verification to penalize the maximal likelihood predictor. Furthermore, the minimal criteria of the penalized maximal likelihood predictor were employed to acquire the quintessential penalty parameter λ values. The GSE57317 and GSE4581 databases were next chosen as the verification cohorts (VCs). Patients were separated into a high- (HR) or low-risk (LR) cohort, depending on the median TC risk score (RS) (**Figure 2**). To further verify the validity of the model, the Kaplan-Meier analysis of survival was performed between both groups. Additionally, the time-dependent receiver operating characteristic (ROC) curve analysis was established to reflect the sensitivity and specificity of predictions (**Figure 3**).

**Figure 4:**
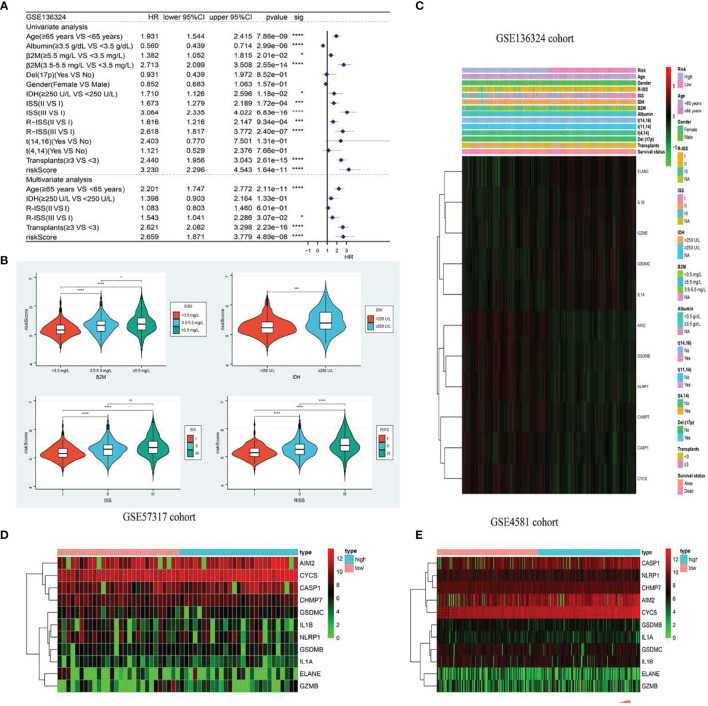
The model is significantly correlated with clinicopathological factors in MM patients and validates survival prediction. **(A)** Univariate (top) and Multivariate(bottom) COX analysis in training cohort. **(B)** The the relationship between clinical features and risk groups in training cohort. **(C–E)**. The heatmap displays results for the clinical. *p < .05; **p < .01, ***p < .001 and ****p < 0.0001.

**Figure 5:**
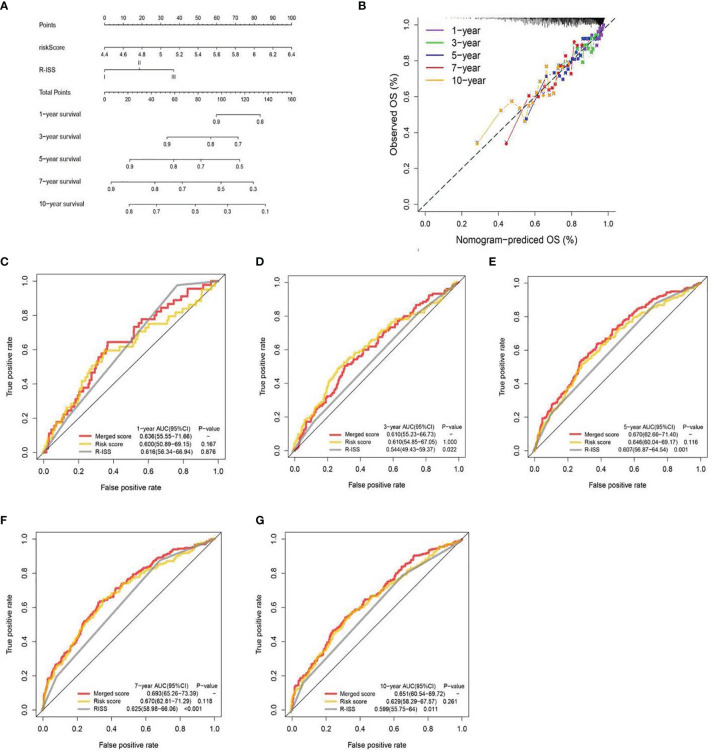
A nomogram was built based on R-ISS and risk score **(A)**, with calibration plot of the nomogram **(B)** and time-dependent receiver operating characteristic (ROC) curves of nomograms were compared based on 1-, 3-, 5-, 7-, and 10-year OS of the cohort **(C–G)**.

The univariate and multivariate Cox analysis for RS and prognostic indicator were performed in TC (**Figure 4**). Moreover, a nomogram was built based on Revised International Staging System(R-ISS) and RS with the consistency index (C index) and the curve of calibration (**Figure 5**).

### Functional Enrichment Analysis

**Figure 6:**
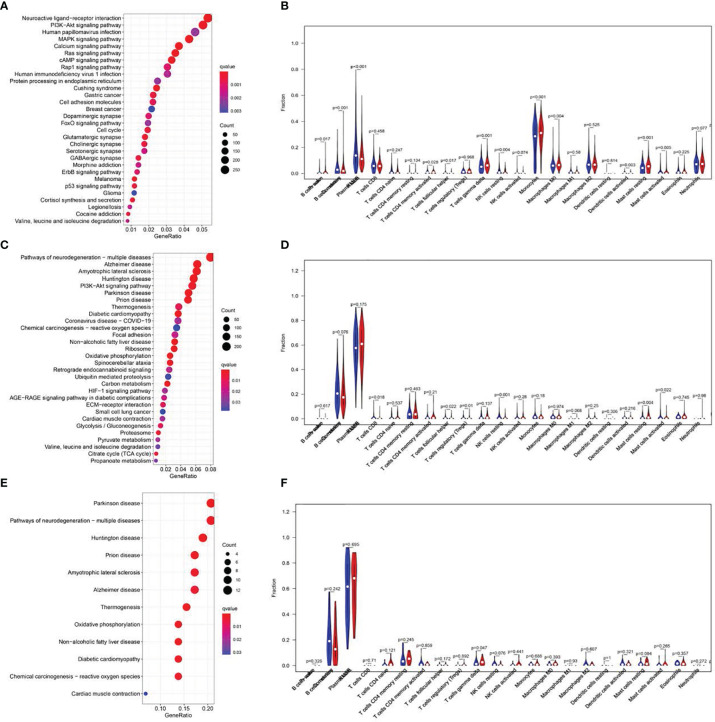
KEGG pathway analysis and correlations of the signature with immune cell infiltration. Top 20 significantly enriched KEGG pathways in the LR group of the training cohort. **(A, C, E)**. Analysis of immune cell infiltration. The blue and red violin represented the IRGPI LR and HR group, respectively. The white points inside the violin implicated median values **(B, D, F)**.

The GSEAv4.0.2 software (http://software.broadinstitute.org/gsea/login.jsp) and c2.cp.kegg.v7.0.symbols gene sets were employed to elucidate the physiological pathways associated with the HR and LR patient cohorts. NOM P-value <0.05 was deemed significant. Additionally, we explored differences in immune cell infiltration landscapes between the two patient populations, using the signature-identified *via* the CIBERSORT algorithm (**Figure 6**).

### Quantitative Real-Time PCR

Bone marrow specimens were accumulated from MM (n=20) and non- hematological malignancy patients (n=20) at the Sun Yat-sen University Cancer Center. Total RNA was extracted with TRIzol (Thermo Fisher Scientific, USA) and corresponding cDNA was synthesized using PrimeScript™ RT Master Mix (Takara Bio, USA), followed by qRT-PCR employing TB Green^®^ Premix Ex Taq (Takara Bio, USA). Intergroup analysis was done with the Student’s t-test (two-tailed). The employed primer sequences are presented in **Table S2**.

### Cell Culture

The MM cell lines U266 and RPMI 8226 were acquired from the American Type Culture Collection (ATCC, Manassas, VA, USA), and maintained in a 37°C humid chamber with 5% CO_2_. RPMI-1640 medium was used with 10% foetal bovine serum and 100 IU/mL of penicillin and streptomycin each (RPMI 1640, FBS and Pen-Strep were from Gibco).

### Cell Viability and Apoptotic Assay

Cell viability was examined *via* Cell Counting Kit-8 assay (CCK-8) (Dojindo, Japan), following kit directions. Doxorubin (DOX, S1208) and Q-VD-Oph (S7311) were acquired from Selleck Chemicals (Houston, TX, USA). U266 and RPMI 8226 cells were grown in 96-well plates with 10000 cells per well in an incubator with 5% CO2 at 37°C for 24 h. Cells were next exposed to the compound at specified concentrations for 48 h, before introduction of CCK-8 kit reagent, followed by a 2 h incubation. Optical density was recorded at 450 nm with a microplate reader. All experiments were done two or more times. In terms of cell apoptosis, compound-treated (specified concentrations for 48 h) U266 and RPMI 8226 cells were lysed and twice rinsed in chilled PBS. Subsequently, the cells underwent FITC-Annexin V/PI staining (KeyGEN, China), following kit directions. The staining was done for 15 min in the dark before detection and quantification of apoptosis using flow cytometry.

### Western Blotting

Cells were treated as specified for 48 h, then collected and centrifuged at 1000 rpm for 5 min to obtain pellets, which were then lysed in RIPA buffer, enhanced with protease and phosphatase inhibitors (PHYGENE, SantaCruz Inc, Dallas, TX, USA), for 1-2 h. The lysates underwent further vortex mixing, followed by sonication in an ice-water bath for 5 min on high for 30 s and 1 min intervals. The resulting solution was spun down at 12,000 rpm for 15 min, and, following total protein quantification, 50 µg protein was boiled with sample buffer at 95 ° for 5 min, and electrophoresed in a 10% sodium dodecyl sulphate-polyacrylamide gel (SDS-PAGE) before transfer to polyvinylidene fluoride (PVDF) membrane. As primary antibodies, we employed anti-β-actin (1:1000; CST, Boston, MA, USA), GSDME (1:1000; Abcam, Britain), and as secondary antibody we employed anti-rabbit IgG H&L (HRP) antibody (Abcam, ab205718).

### Statistical Analysis

Inter-group analyses were done using Student’s t test or one-way ANOVA. Categorial data was analyzed using the Chisq or Fisher exact test, SPSS software version 25 (IBM Corporation, Armonk, NY, USA). A two-sided *P*-value <0.05 was deemed significant. All data analyses employed the R software (version 3.6.3 for Windows, http://www.R-project.org). All experiments were done thrice, and the resulting data displayed as means ± SD.

## Results

### Patient Selection and Demographics

Overall, 986 MM patients with relevant RNA profile and corresponding clinical information from the GEO database were included in our analysis (**Table S1**). To identify PRGs related to MM prognosis, we specified the GSE136324 cohort(n=866) as the TC, and the GSE57317 (n=55) and GSE4452 (n=65) populations as the VCs. **Figure 1** summarizes our research design.

### Establishment of a Prognostic PRGs Signature

We identified 57 PRGs with matching transcript profiles from all three patient populations. Using univariate analysis, we further identified 19 PRGs that relate to MM overall survival (OS) from the GSE136324 cohort (P<0.05) (**Figure 2A**). Next, we employed LASSO analysis to generate a PRG signature that accurately predicts MM prognosis. According to our penalized maximal likelihood predictor of 100000 bootstrap replicates, a profile of 11 PRGs (namely, *AIM2, CASP1, ELANE, GSDMB, GSDMC, IL1B, NLRP1, GZMB, IL1A, CHMP7*, and *CYCS*) was generated, carrying a minimal criteria optimal λ value (**Figures 2B, C**). **Figure 2E** illustrates the tumor mutation profile of the 11 PRGs. The model equation is provided below:

RS = 0.07 X *AIM2* levels + 0.12 X *CASP1* levels - 0.06 X*ELANE* levels + 0.06 X *GSDMB* levels - 0.07 X*GSDMC* levels - 0.05 X *IL1B* levels + 0.31 X *NLRP1* levels - 0.02 X *GZMB* levels - 0.16 X*IL1A* levels + 0.25 X *CHMP7* levels + 0.16 X *CYCS* levels.

The TC patients were assigned to high-risk (HR, n=433) or low-risk (LR, n=433) populations, according to the median threshold. LR patients experienced remarkably prolonged OS time, relative to HR patients (P<0.05) (**Figure 3A**). Next, we tested the model reliability with time-dependent ROC curves. The TC AUCs for the 1-, 3-, 5-, 7-, and 10-year OS were 0.626, 0.621, 0.654, 0.674, and 0.637, respectively (**Figure 3B**). The VC RS was computed based on the aforementioned formula, and the median TC RS was used as the threshold. The curves were next applied to the internal VC, and the subsequent AUCs were 0.778 and 0.715, 0.709 and 0.628, as well as 0.645 and 0.769 for the 1-, 3- and 5-year OS in the GSE57317 and GSE4581 populations, respectively. The PRG signature exhibited an elevated OS predictive accuracy in both VCs. Moreover, the HR patient OS was remarkably worse than the LR patient OS, as evidenced by the Kaplan-Meier analysis. Given these data, our model demonstrated persistent superior performance.

### RS Profile and Prognosis-Based Analysis of Clinicopathological Features

Based on our uni- and multivariate analyses examining RS and other prognostic factors, the RS serves as a stand-alone OS prognostic marker in the GSE136324 population (**Figure 4A**). We further assessed the associations between RS profile and clinicopathological features. We observed marked differences between beta2-Microglobulin (β2-MG) and lactate dehydrogenase (LDH) levels, as well as the International Staging System (ISS) and Revised ISS (R-ISS) (**Figure 4B**). **Figures 4C–E** illustrates the correlations between clinicopathological characteristics and both datasets as heatmaps, whereby each dataset exhibits a slightly altered, yet consistent, gene expression.

### Construction of the Estimation Nomogram

We generated a nomogram to collectively display data from the R-ISS stage and PRG profile of the TC (**Figure 5A**). Calibration curve and consistency index (C-index) assessed the predictability of our model. The merged score AUC was higher than the R-ISS stage, indicating the significance of the nomogram in enhancing OS estimation (**Figures 5B–E**).

### Gene Set Enrichment Analyses (GSEA) and Correlation of RS With Immune Status

GSEA was utilized to explore the physiological roles and related pathways associated with RS. Based on our analysis, a majority of PRGs participated in cell proliferation, metabolism, and immune response pathways (**Figures 6A, C, E**). We next employed CIBERSORT (16) to estimate the differences between 22 distinct forms of tumor-infiltrating immune cells between the LR and HR patients. Relative to HR patients, we demonstrated a marked reduction in tumor infiltration, particularly that of monocytes and B cells in the LR group in the TC (P<0.001, **Figure 6**)

### Verifying the Expressions of 11 PRGs in Specimens

**Figure 7:**
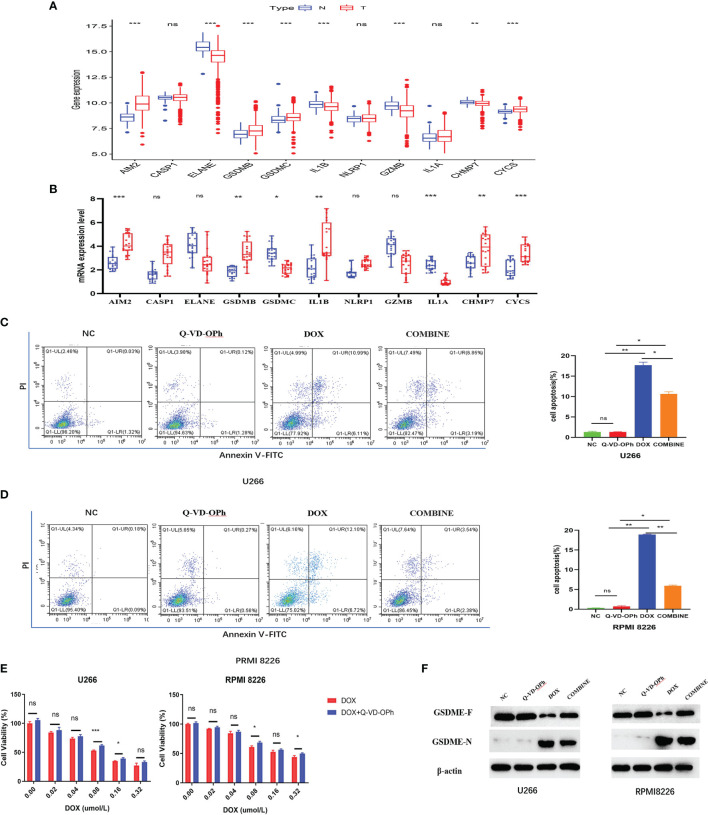
**(A)** The expression difference of PRGs in normal tissues and tumor tissues in GSE118985. **(B)** qRT-PCR analysis of 11 genes expression in tissues. The flow cytometry results showed DOX induces cell apoptosis *via* the activation of pyroptosis in U266 cells **(C)** and RPMI 8226 cells **(D)**, treated as indicated. **(E)** The CCK-8 experiment was used to detect the cell viability treated by DOX or Q-VD-OPh as indicated. The maker was determined by Western blot analysis **(F)** showed the protein expression of the GSDME-F and GSDME-N with β-actin as loading control. The cells were treated by DOX or Q-VD-OPh or in combination at 48 hours treated as indicated. Data were presented as the mean ± SD. Experiments were performed at least three times. *p <. 05; **p < .01, ***p < .001, ns, P > 0.05.

Validation of the expression of 11-PRGs in normal tissues and tumor tissues in GSE118985 and our cohort are shown in **Figures 7A, B**. Based on our results, *AIM2, CASP1, GSDMB, NLRP1, CHMP7*, and *CYCS* were upregulated, whereas, *ELANE, GSDMC, IL1B, GZMB*, and *IL1A* were downregulated in MM specimens versus controls (**Figure 7B**).

### Q-VD-OPH Weakens Doxorubin (DOX) Sensitivity in MM Cells

First, we assessed MM cells (U266 and RPMI 8226) viability after exposure to DOX and Q-VD-OPH alone or in combination. MM cells were incubated with varying doses of DOX (0 μM, 0.02 μM, 0.04 μM, 0.08 μM, 0.16μM and 0.32μM) and Q-VD-OPH (40μM) in combination or alone for 48h, and cell viability was examined with CCK8. Based on our results, the combined treatment greatly enhanced cell viability, relative to DOX alone in both cell lines (**Figure 7E**).

### Q-VD-OPH Inhibits DOX-Induced MM Cell Apoptosis

Using Annexin V -FITC/PI-staining and flow cytometry, we analyzed U266 and RPMI 8226 cell apoptosis after treatments with DOX (0.08μM) and Q-VD-OPH (40μM) alone or in combination for 48h. We demonstrated that DOX significantly induced cell apoptosis while Q-VD-OPH significantly decreased DOX-induced cell apoptosis in both cell lines (**Figures 7C, D**). These results demonstrate that DOX stimulated apoptosis by activating pyroptosis in U266 and RPMI 8226 cells.

Doxorubin (DOX), a common chemotherapeutic agent, induces pyroptosis *via* caspase-3-induced slicing of GSDME (GSDME-N) (17). Q-VD-OPH, a pan-caspase suppressor, forms covalent bonds with and permanently sequesters caspase-3 (18).So we detected the GSDME- N and GSDME- F protein expressions in two cell lines incubated with DOX and Q-VD-OPH alone or in combination western blot analysis (**Figure 7F**) to confirm that DOX-induced pyroptosis is drastically reduced by Q-VD-OPH in MM cells. Collectively, these evidences suggest that DOX- induced apoptosis, which play an essential function in cancer treatment, is related to pyroptosis.

## Discussion

Pyroptosis, a novel form of programmed cell death, serves essential roles in both tumor formation and therapeutic pathways. Pyroptosis occurs in cells infected by pathogens, where a large number of inflammatory factors are released, resulting in strong a immunological response, and induction of the body’s inflammatory response (19). Healthy cells, stimulated by pyroptosis-related inflammatory factors, often turn tumorous. Alternately, inducing tumor cell pyroptosis may achieve anti-tumor effects (20). Pyroptosis plays distinct roles in numerous cancers, and multiple prognostic PRG signatures have been constructed for use in different kinds of cancers (20–24). However, the potential value of PRG in MM have not been elucidated.

Herein, we assessed the expression profile of 57 PRGs from prior literatures. We compared the PRG signature and its possible physiological activities against the clinical and transcriptome data of MM samples in the GEO sets, using both Cox and LASSO analyses. Based on our analysis, we identified an 11 PRG gene signature, namely, *AIM2, CASP1, ELANE, GSDMB, GSDMC, IL1B, NLRP1, GZMB, IL1A, CHMP7*, and *CYCS*, that is related to MM patient prognosis. The absent in melanoma 2 (AIM2) protein resides in the cytoplasm and acts as a sensor for double-stranded DNA. Upon detection, it interacts with the apoptosis-related speck-like protein that contains CARD (ASC) and procaspase-1, and forms a multi-protein AIM2 inflammasome, which is reported to have both anti- and pro-tumorigenesis activities (25, 26). Also known as a member of the inflammasome complex, the pyrin domain-harboring the protein 3 (NLRP3) inflammasome contributes to inflammation, and regulates cancer pathogenesis by modulating immune response, cell death, and proliferation (27, 28). Based on our analysis, these appear to be cancer-inducing genes, which provide some insights into further investigations. GasderminD (GSDMD), in the inflammasome, activates CASP1, a member of the cysteinyl aspartate protease (caspase, or CASP) gene family to induce pyrexia (29). This gene family is reported to be intricately linked to cancer immune infiltration, and may be used as a target for immunotherapy (30). Released by neutrophils, neutrophil elastase (ELANE) attenuates growth of primary tumors and, in turn, produces CD8 + T cell-regulated distant effects that target remote metastases. Studies revealed that ELANE kills cancer cells using diverse genes, but has the least toxicity to non-cancer cells. As such, it holds great potential in a wide range of anti-cancer therapies (31).

GSDMB and GSDMC belong to the Gasdermin (GSDM) protein family. They possess dual roles in cancer, depending on their association with tumor cells and TME, and may or may not involve cell death activities (i.e. be tumor-promoting or anti-tumor) (32, 33). IL1A and IL1B are key cytokines that regulate inflammation and influences TME, thus promoting the origin and progression of various cancers. They are strongly associated with tumor cell proliferation and treatment of various tumors (34–36). Granzyme B (GzmB) is typically cleaved following Aspartic acid and triggers the activation cascade of caspases responsible for apoptosis. It is an enzyme necessary for the lysis of target cells in a cell-mediated immune response. Emerging studies suggest its strong relation with tumor progression and regression, and immune microenvironment (37–39). Charged multivesicular body protein 7 (CHMP7) forms a complex with ESCRT III in the ESCRT system, and initiates the degradation of cell surface receptors (40, 41). In addition, CHMP7 is highly expressed in certain hematological malignancies, which is consistent with our research (42). Cytochrome c somatic (CYCS) is associated with mitochondrial dysfunction and autophagy defects, and it also has potential mechanism in tumor cell proliferation and apoptosis (43, 44).

Our model demonstrates enhanced OS in LR versus HR patients in the TC and VCs. We predicted OS using the nomogram and employed the time-related ROC curves to test the model. The overall results revealed that this model possesses higher prognostic value than other staging systems. We further analyzed the physiological activities related to the risk score. Our KEGG analyses revealed that the differentially regulated PRGs primarily participated in immune responses and inflammatory cell chemotaxis. Hence, we speculated that pyroptosis modulates the composition of TME.

Pyroptosis is initiated *via* activation of multiple networks. Among the classical inflamassome networks is the activation of Caspase-1, which nicks cytoplasmic gasdermin D (GSDMD) such that the N-terminal forms a transmembranal pore to induce apoptosis. Another network involves the atypical inflammasome pathway, which is induced by lipopolysaccharide (LPS) directly binding to Caspase-4/5. A third mechanism involves the caspase-3-based apoptotic network, which cleaves gasdermin E (GSDME-N) to generate a transmembranal pore to initiate apoptosis (45, 46).. Some chemotherapeutic drugs work by activating pyroptosis to inhibit tumor progression (9).

Doxorubicin (DOX) is widely used as the main pharmaceutical intervention for MM, which is not only usually prescribed in combination with other adjuvant drugs (47–49),but also found to be a inducer of pyroptosis *via* caspase-3-induced slicing of GSDME (GSDME-N),which could trigger pyroptosis (17, 50). In recent years, studies on DOX-induced pyroptosis revealed a heavy involvement of caspase-3-mediated GSDME activation, which suggests that GSDME may be a potential target for drug research (17, 50, 51). However, there are still few studies on the mechanism of DOX-induced pyroptosis of myeloma cells. Q-VD-OPh, a wide-ranging caspase inhibitor, further confirmed the involvement of caspase-dependent apoptosis and permanently sequesters numerous caspases (caspase-1, caspase-3, and caspase-7 to caspase-12), and it showed no cell toxicity even at remarkably high concentrations (18, 52). In order to verify the pyroptotic function in MM treatment, we employed DOX to induce pyroptosis in two MM cell lines. Upon Q-VD-OPh addition, the DOX-induced cell death was reduced, as evidenced by flow cytometry, thus indicating that DOX-induced cell death may be related to pyroptosis. Furthermore, the protein levels of cleaved GSDME in MM cells treated by DOX verified the fact that DOX triggers pyroptosis. In addition, after treatment with Q-VD-OPh, the levels of GSDME-N induced by DOX decreased, suggesting that Q-VD-OPh inhibits DOX-induced pyroptosis to some extent. The findings suggest that gene targets related to pyroptosis may provide novel insights for future anti-MM therapy.

## Conclusions

In conclusion, we established a novel PRG-based signature for MM patient prognosis. Our analysis revealed that pyroptosis, to some extent, influences the physiological activities of tumors *via* immunologic modulation. We verified our results using both external datasets and clinical samples. In future investigations, the potential mechanism of scorch death in MM needs to be explored.

## Data Availability Statement

Publicly available datasets were analyzed in this study. This data can be found here: http://www.ncbi.nlm.nih.gov/geo/.

## Ethics Statement

The studies involving human participants were reviewed and approved by Ethics Committee of Sun Yat-sen University Cancer Center. The patients/participants provided their written informed consent to participate in this study.

## Author Contributions

Study concept and design, HW and RS. Data collecting, SL, SB, and BF. Statistical analysis, HW, RS, and SL. Figure and tables preparation, HW, RS, SL, SB, and BF. Writing-original draft, HW, RS, and SL. Data and tables inspection and validation, SB and BF. Project administration, YL and RL. Work supervision and writing-review and editing, YL and RL. Funding acquisition, YL. Critical revision of the manuscript for important intellectual content, all authors.

## Funding

This work was supported by the following funds, National Natural Science Foundation of China under grant (Number 30471976 and 81272620) and Science and Technology Projects of Guangdong Province under grant (Number 2016A020215086).

## Conflict of Interest

The authors declare that the research was conducted in the absence of any commercial or financial relationships that could be construed as a potential conflict of interest.

## Publisher’s Note

All claims expressed in this article are solely those of the authors and do not necessarily represent those of their affiliated organizations, or those of the publisher, the editors and the reviewers. Any product that may be evaluated in this article, or claim that may be made by its manufacturer, is not guaranteed or endorsed by the publisher.
